# Janus Kinase Inhibitors As Emerging Therapy in the Treatment of Psoriatic Arthritis: A Narrative Review of Efficacy, Safety, and Future Perspective

**DOI:** 10.7759/cureus.92166

**Published:** 2025-09-12

**Authors:** Marian Barsoum, Muram Mohamed, Alameen Ibrahim, Ahmed Khalfalla, Liena Elbashir

**Affiliations:** 1 Medical Education and Simulation, Faculty of Medicine, Assiut University, Assiut, EGY; 2 Internal Medicine, The Combined Research Foundation, Okemos, USA; 3 General Practice, Nile College, Khartoum, SDN; 4 Medicine, International University of Africa, Khartoum, SDN; 5 Medical Education and Simulation, Faculty of Medicine, University of Gezira, Wad Madani, SDN; 6 Internal Medicine, University of Medical Sciences and Technology, Khartoum, SDN; 7 Internal Medicine, Faculty of Medicine, University of Khartoum, Khartoum, SDN

**Keywords:** acr response, adalimumab, efficacy, filgotinib, jak inhibitors, pasi, psoriatic arthritis, safety, tofacitinib, upadacitinib

## Abstract

Psoriatic arthritis (PsA) is a chronic, immune-mediated inflammatory disorder that affects peripheral joints, entheses, and the skin, driven by the dysregulation of key cytokine pathways and often necessitating targeted immunomodulatory therapy for optimal disease control. While biologic therapies targeting tumor necrosis factor (TNF), interleukin-17 (IL-17), and interleukin-23 (IL-23) pathways have transformed PsA management, limitations in efficacy, tolerability, and patient access persist. Janus kinase (JAK) inhibitors represent an emerging class of targeted therapies, offering a distinct mechanism of action by disrupting intracellular signaling pathways involved in inflammation. This narrative review summarizes recent clinical trial data and real-world evidence on the efficacy and safety of JAK inhibitors, with particular attention to their comparative performance against adalimumab and other biologic classes. To facilitate patient selection and monitoring, important safety factors such as cardiovascular risk, cancer, and venous thromboembolism are contextualized within the most recent guidelines. Beyond summarizing outcomes, we critically examine the clinical implications of integrating JAK inhibitors into PsA treatment algorithms. As the therapeutic landscape continues to evolve, further long-term studies are essential to establish the optimal role of JAK inhibitors in clinical practice and clarify their position within the broader treatment framework for PsA. These findings can help guide treatment choices and support decisions made with patients.

## Introduction and background

**Figure 1:**
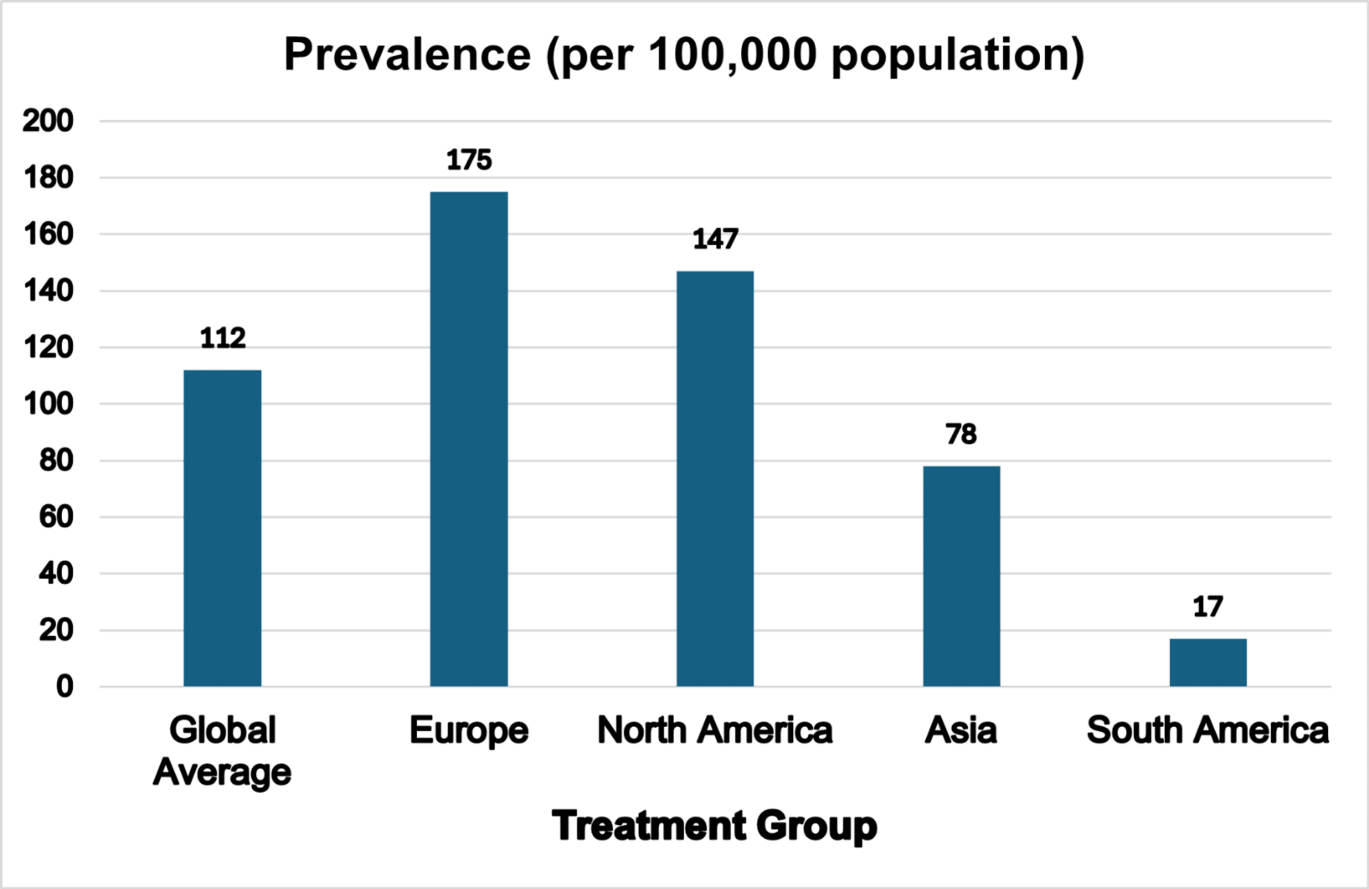
Psoriatic Arthritis (PsA): Continental Prevalence and Global Comparison This figure highlights geographic variation in PsA prevalence, with notably higher rates in Europe and North America, reflecting differences in genetics, diagnostics, and healthcare access. These trends highlight the need for region-specific treatment strategies and equitable access to therapies like Janus kinase (JAK) inhibitors. Credit: This figure has been created by the authors using data from Lembke et al. [3], visualized in Microsoft Excel (Microsoft Corp., Redmond, WA, USA).

Psoriatic arthritis (PsA) is a form of inflammatory arthritis associated with psoriasis, typically presenting with a combination of extra-articular manifestations such as dactylitis and enthesitis, as well as inflammatory bowel disease, uveitis​, and psoriatic plaques​ [1]. PsA is often underdiagnosed due to the absence of internationally accepted diagnostic criteria and inconsistent study methods, making its true prevalence difficult to determine. Gladman et al.’s study suggests that 2-3% of the general population has inflammatory arthritis, with 6% to 42% of people with psoriasis affected [2]. According to a meta-analysis by Lembke et al., global PsA prevalence is around 112 individuals per 1000 population. Regional variation is notable,​ as illustrated in Figure 1 [3].

PsA has many genetic associations, which include HLA-B*08:01, HLA-B*27:05, HLA-B*38:01, HLA-B*39:01, HLA-B*44:02, HLA-B*57:01, and HLA-C*06:02 [4]. Among these, HLA-B27:05 shows the strongest associations. HLA-B27:05 is particularly linked to axial disease and is also found in other spondyloarthritides such as ankylosing spondylitis, suggesting a shared pathogenic mechanism [5]. In those who are genetically predisposed, environmental factors such as infections, trauma, stress, obesity, and smoking have all been implicated to a lesser extent in the triggering of PsA. The genetic contribution to such diseases has traditionally been examined through twin studies. A concordance rate for psoriasis of 20% to 64% was observed in monozygotic twins, according to a study by Queiro et al., indicating that approximately 70% of the population variance in susceptibility to psoriasis can be attributed to genetic factors, which also applies to PsA [5]. The interleukin-23/interleukin-17 (IL-23/IL-17) axis in the immune system is considered a key driver, with IL-23 from dendritic cells and macrophages activating resident T cells. This activation results in increased production of interleukin-17A (IL-17A), interleukin-22 (IL-22), and tumor necrosis factor-alpha (TNFα), which in turn promotes extra-articular manifestations, bone erosion, and abnormal new bone formation [6]. Moreover, multiple cytokines, including IL-23, IL-6, and type I interferon, signal through the Janus kinase-signal transducer and activator of transcription (JAK-STAT) pathways [7]. This positions the JAK-STAT axis as a critical upstream regulator of PsA inflammation, making it a rational target for therapeutic intervention.

In light of this, managing PsA is challenging due to its complex causes, including genetic factors and varied immune system pathways. Comorbidities and extra-articular symptoms further complicate care. Early diagnosis and treatment are pivotal to improving the quality of life and preventing long-term complications. Without treatment, PsA can lead to disability and increase the risk of comorbidities such as cardiovascular disease, diabetes, thyroid disorders, obesity, and depression [8,9].

While PsA has no cure, treatment focuses on controlling inflammation and slowing disease progression. Historically, options were limited to cyclosporine, methotrexate (MTX), and phototherapy [10]. The newer biologic agents, including JAK inhibitors, TNF inhibitors, and phosphodiesterase-4 (PDE4) inhibitors, have shown greater efficacy in clinical trials [10,11].

As our understanding of PsA pathogenesis has advanced, treatment strategies have evolved in parallel. Recent developments include JAK inhibitors, small synthetic molecules that inhibit the JAK-STAT signaling pathway, thereby reducing the production of proinflammatory cytokines. The selectivity for specific JAK isoforms distinguishes two generations of these agents. First-generation drugs, such as tofacitinib, baricitinib, ruxolitinib, and peficitinib, exhibit reduced JAK selectivity, whereas second-generation inhibitors such as filgotinib and upadacitinib demonstrate greater selective JAK-1 selectivity [12].

The JAK-STAT pathway regulates inflammatory and immune responses through gene expression and DNA transcription. It involves cell surface receptors, JAKs (JAK1, JAK2, JAK3, TYK2), and STAT proteins. Upon cytokine binding, such as ILs or interferons, JAKs are activated, leading to STAT phosphorylation and downstream signaling. Over the last decade, JAK inhibitors have played a significant role in managing PsA, particularly for patients with inadequate response to conventional disease-modifying antirheumatic drugs (DMARDs). In addition to PsA, JAK inhibitors such as tofacitinib and upadacitinib are also approved for other immune-mediated conditions, including rheumatoid arthritis (RA) and ulcerative colitis, further supporting their role in modulating systemic inflammation. Some patients prefer JAK inhibitors due to their oral administration and relative affordability [13].

**Figure 2:**
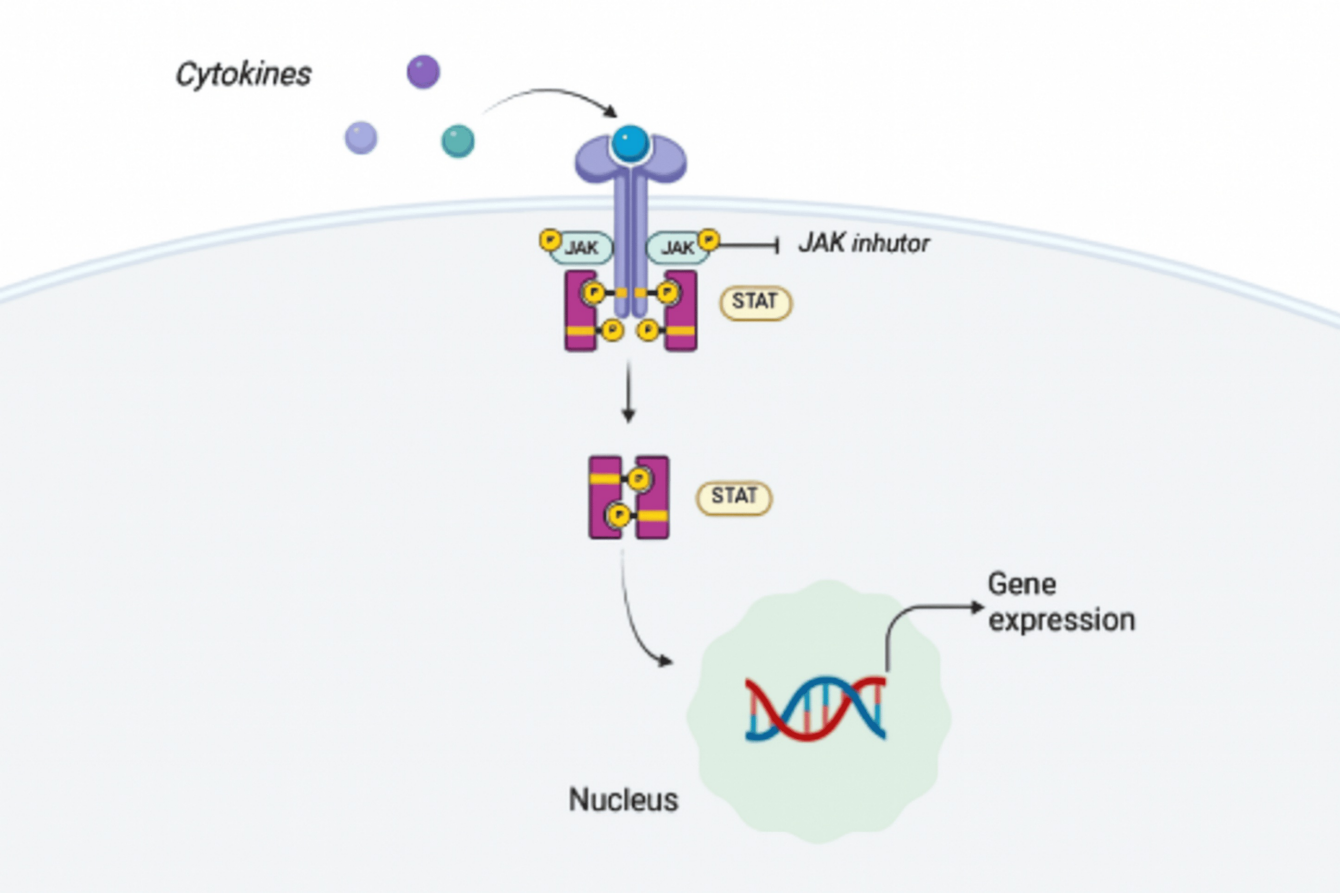
JAK-STAT Signaling Pathway This figure illustrates how JAK inhibitors block cytokine-mediated activation of the JAK-STAT pathway, preventing downstream gene transcription involved in inflammation. This targeted disruption supports their therapeutic role in PsA. Credit: This figure has been created by the authors using data from Shawky et al., Lin et al., and Chikhoune et al. [12-14] with visual elements generated using BioRender.com (BioRender, Toronto, ON, Canada). JAK: Janus kinase; STAT: single transducer and activator of transcription; PsA: psoriatic arthritis

Phosphorylated STAT proteins migrate to the nucleus, where they can perform their function as transcription factors and gene expression regulators. This process is central to the JAK-STAT signaling cascade, which is illustrated in Figure 2. The aberrant JAK-STAT pathway is responsible for immune system dysregulations and different types of cancer [12-14]. Therefore, blocking the JAK-STAT can control undesirable immune system dysfunctions.

This review aims to critically evaluate the efficacy of JAK inhibitors in PsA, focusing on treatment response across disease domains, the impact of dosing strategies, and the alignment between clinical improvement and composite disease activity scores. By addressing these gaps, we seek to clarify the therapeutic role of JAK inhibition and inform future research and clinical decision-making.

## Review

Methodology

A targeted literature search was performed using PubMed covering publications from January 2015 to June 2025. Search terms included combinations of “psoriatic arthritis,” “JAK inhibitors,” “tofacitinib,” “upadacitinib,” “adalimumab,” “TNF inhibitors,” “efficacy,” “safety,” and “clinical outcomes.”

The review included peer-reviewed original research articles, full-text clinical trials, observational studies, systematic reviews, and meta-analyses published in English, focusing on adult patients (≥19 years) diagnosed with PsA. Studies involving pediatric populations, animal models, non-English publications, or abstracts lacking full text were excluded.

Although Rayyan (Rayyan Systems Inc., Cambridge, MA, US) was initially used to organize references, no formal systematic screening protocol was applied. Manual reference checks were also conducted to identify additional relevant studies. Titles and abstracts were reviewed for thematic relevance, followed by full-text appraisal. One reviewer conducted the initial screening and data extraction, while a second reviewer independently verified the extracted outcomes to minimize selection bias. Clinical outcomes such as American College of Rheumatology (ACR) response rates, Psoriasis Area and Severity Index (PASI) scores, patient-reported outcomes such as the Psoriatic Arthritis Impact of Disease 9-item questionnaire (PsAID9) and the Short Form-36 Health Survey (SF-36), as well as enthesitis indices including the Leeds Enthesitis Index (LEI) and the Spondyloarthritis Research Consortium of Canada (SPARCC) Enthesitis Index were extracted and qualitatively discussed. These measures were chosen for their relevance in evaluating joint inflammation, skin involvement, quality of life, and enthesitis burden in PsA populations.

Findings were organized thematically to highlight immunologic mechanisms, treatment efficacy, safety concerns, and comparative insights with TNF inhibitors. No formal meta-analysis or systematic screening protocol was employed. There was no conflict of interest to be disclosed.

Efficacy and safety

Using JAK inhibitors in RA patients produced significant therapeutic benefits and has a recognized safety profile. Recent clinical trials have explored JAK inhibitors in PsA, demonstrating encouraging outcomes. These findings led to approval of tofacitinib as the first JAK inhibitor for PsA treatment, followed by upadacitinib in 2021 [15].

A clinical trial conducted by Muensterman et al. showed that for some endpoints, the 15 mg dose of upadacitinib was adequate to provide a maximal response; however, higher exposures, attained with the 30 mg dose, resulted in significant improvements for more demanding measures. Although the exposure-response relationship correlates with enhanced efficacy, it concurrently increases the likelihood of serious safety events [15], as demonstrated in Table 1.

**Table 1:** The Clinical Efficacy of Upadacitinib in Comparison to Placebo in Psoriatic Arthritis ACR50: American College of Rheumatology 50% improvement criteria; ACR70: American College of Rheumatology 70% improvement criteria; sIGA: static Investigator Global Assessment of Psoriasis; q.d.: once daily Values are presented as median with 90% prediction intervals (PI). Credit: This table has been adopted from Muensterman et al. [15], which is an open-access article distributed under the terms and conditions of a CC BY-NC-ND 4.0 license.

Clinical efficacy response	Placebo	Upadacitinib 15 mg q.d.	Upadacitinib 30 mg q.d.
ACR50 at week 12	11% (7-15%)	37% (32-43%)	45% (39-50%)
ACR70 at week 12	2% (1-3%)	14% (10-19%)	21% (16-26%)
sIGA 0/1 at week 16	10% (7-14%)	40% (34-45%)	49% (43-55%)
sIGA 0/1 at week 24	11% (8-16%)	40% (36-47%)	49% (42-55%)

According to a meta-analysis done by Wang et al., JAK inhibitors' efficacy in treating PsA is largely dependent on the type of inhibitor and the length of treatment. ACR 20, 50, and 70 response rates indicated that selective JAK1 inhibitors significantly increased disease activity, but combined JAK1/JAK3 inhibitors had no statistically significant impact. It wasn't until 16 weeks into the treatment that these gains in ACR scores were noticed. Only dual JAK1/JAK3 inhibitors enhanced the mental components (SF-36 MCS), with the strongest outcomes shown in 12 weeks. The physical components (SF-36 PCS) improved with all JAK inhibitors, which reflects a better quality of life throughout the study. The Disease Activity Index for Psoriatic Arthritis (DAPSA) scores did not significantly improve for any inhibitor type, suggesting that while symptoms may improve, deeper inflammation might not be well controlled [10].

In a clinical trial by Cantini et al., enthesitis resolution was evaluated using two validated tools: the LEI, which evaluates tenderness at six sites, and the SPARCC Enthesitis Index, which examines 16 anatomical locations. Patients receiving upadacitinib 15 mg showed significantly greater improvement than those on placebo across all time points. By week 24, 59.8% of upadacitinib-treated patients were enthesitis-free per LEI, compared to 38.0% in the placebo group. SPARCC scores similarly favored upadacitinib (50.6% vs. 31.5%), with all differences statistically significant (nominal P < 0.0001), except SPARCC at week 12 (P = 0.0002). After switching from placebo to upadacitinib at week 24, LEI resolution rates rose to 55.6% by week 36 and 68.2% by week 56, closely matching outcomes in the continuous treatment group [16]. However, the trial did not assess whether enthesitis resolution translated into broader functional improvement or reduced disease burden, limiting its direct clinical applicability and leaving the impact on patients’ daily lives unclear.

**Figure 3:**
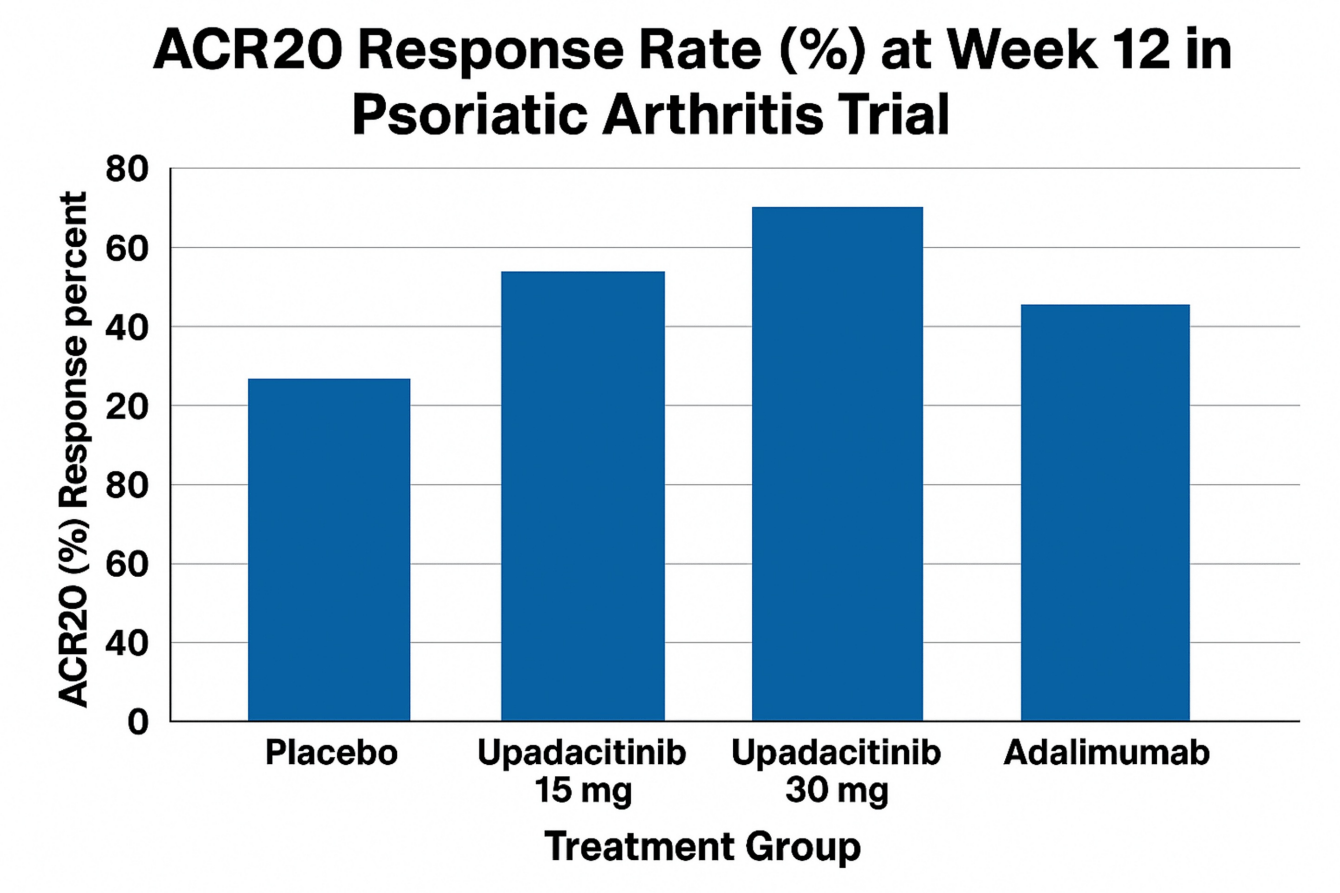
The Figure Shows ACR20 Response Rates for Upadacitinib 15 mg, Upadacitinib 30 mg, Adalimumab, and Placebo Drug This figure shows week 12 ACR20 response rates in active PsA (n = 1,704) from the McInnes et al. phase 3 trial. Patients were randomized 1:1:1:1 to upadacitinib 15 mg (n = 426), upadacitinib 30 mg (n = 426), placebo (n = 426), or adalimumab 40 mg biweekly (n = 426). ACR20 rates were 36.2% with placebo, 70.6% with upadacitinib 15 mg, and 78.5% with upadacitinib 30 mg (P < 0.001 vs. placebo), and 65.0% with adalimumab. The 30 mg dose was superior to adalimumab (Δ 13.5 pp; 95% CI 7.5-19.4). Δ: difference; pp: percentage points; ACR20: American College of Rheumatology 20% improvement criteria; PsA: psoriatic arthritis Credit: This figure has been created by the authors using data from McInnes et al. [17], visualized in Excel (Microsoft Corp., Redmond, WA, US).

In a phase 3 trial by McInnes et al., upadacitinib (15 mg and 30 mg) was evaluated against placebo and adalimumab in patients with active PsA. Both upadacitinib doses showed significantly greater efficacy than placebo at week 12. While the 30 mg dose met criteria for statistical superiority over adalimumab, the 15 mg dose did not, despite a numerically higher response rate. The formal statistical study had to end there since the 15-mg dose did not satisfy the predetermined standards for superiority over adalimumab. This means that even if the data appeared positive, a statistical conclusion could not be drawn for many other secondary health outcomes [17], as demonstrated in Figure 3.

According to a clinical trial conducted by Orbai et al., at week 16, filgotinib produced clinically meaningful improvements in overall disease impact (PsAID9) and physical function (SF-36 PCS) compared with placebo, without a significant change in mental health (SF-36 MCS), as demonstrated in Table 2 [18].

**Table 2:** Mean Change in the PsAID9 and SF-36 (PCS, MCS) Severity Scales PsAID9: Psoriatic Arthritis Impact of Disease; PCS: physical component summary; MCS: mental component summary; SF-36: Short Form-36 Health Survey Values are presented as mean change from baseline. Credit: This table has been adapted from Orbai et al. [18], which is an open-access article distributed under the terms and conditions of a CC-BY-NC license.

	Filgotinib mean change (n=65)	Placebo mean change (n=66)	P-value
PsAID9 total score at week 16	-2.3 (1.8)	-0.8 (2.2)	<0.0001
PCS	+7.4 (6.6)	+2.4 (6.6)	<0.0001
MCS	+4.3 (8.3)	+3.2 (9.2)	0.4128

According to Kivitz et al.'s post hoc analysis of the clinical experiment, at month three in patients receiving low (≤15 mg/week) or high (>15 mg/week) doses of MTX, tofacitinib dosages of 5 mg and 10 mg were more effective than a placebo. Unexpectedly, the 5 mg tofacitinib dose appeared to be more effective in people taking a higher dose of MTX, whereas the 10 mg tofacitinib dose was generally more beneficial in patients using a lower dose of MTX [19], as demonstrated in Table 3. These findings suggest a potential interaction between MTX background dosing and tofacitinib response, which may inform individualized treatment strategies.

**Table 3:** ACR and PASI Responses to Tofacitinib Versus Placebo in Psoriatic Arthritis by Methotrexate Dose ACR20: American College of Rheumatology 20% improvement criteria; ACR50: American College of Rheumatology 50% improvement criteria; ACR70: American College of Rheumatology 70% improvement criteria; PASI50: Psoriasis Area and Severity Index 50% reduction; PASI75: Psoriasis Area and Severity Index 75% reduction; MTX: methotrexate Credit: This table has been created by the authors in APA style using data from Kivitz et al. [19], which is an open-access article distributed under the terms and conditions of a Creative Commons license.

	Tofacitinib 5 mg BID	Tofacitinib 5 mg BID	Tofacitinib 10 mg BID	Tofacitinib 10 mg BID	Placebo	Placebo
	MTX dose ≤ 15 mg/week	MTX dose > 15 mg/week	MTX dose ≤ 15 mg/week	MTX dose > 15 mg/week	MTX dose ≤ 15 mg/week	MTX dose > 15 mg/week
ACR20	47.4%	52.4%	54.9%	51.8%	26.3%	28.8%
ACR50	27.6%	35.7%	39.3%	25%	11.3%	15.3%
ACR70	12.9%	24.3%	19.7%	8.9%	5.3%	10.2%
PASI50	41.7%	63%	66.7%	66.7%	25.3%	35%
PASI75	29.8%	41.3%	45.7%	44.4%	13.1%	17.15%

JAK inhibitors’ adverse events correlate closely with both the JAK isoform blockade and underlying patient factors. Broad‐spectrum (pan-JAK) agents such as tofacitinib and baricitinib, which inhibit JAK1 and JAK2 (and JAK3 for tofacitinib), carry the highest observed rates of venous thromboembolism and major cardiovascular events [12]. Tofacitinib is particularly linked to a higher incidence of herpes zoster, especially in individuals over 50 and of Asian descent. Additionally, it is associated with dyslipidemia and elevated liver enzymes and requires careful monitoring of all agents [20]. Baricitinib, another non-selective JAK inhibitor studied primarily in RA, reduces neutrophil count and has been associated with thrombotic events such as deep vein thrombosis and pulmonary embolism [20,21].

However, more selective JAK1 inhibitors (e.g., upadacitinib) tend to spare JAK2-driven hematopoiesis, translating into lower incidences of anemia, thrombocytopenia, and thrombotic complications, although long-term malignancy data remain limited across all agents [22]. Filgotinib, another selective JAK1 inhibitor, is currently under phase 3 evaluation and has shown no serious adverse effects in 16-week trials [23].

In the light of this, younger patients (<60 years) without a history of thrombosis or cancer and with controlled comorbidities often tolerate pan-JAK therapy well, provided they undergo baseline venous thromboembolism risk assessment, lipid panel evaluation, and cancer screening. In contrast, older adults or those with prior thrombotic or malignant conditions derive greater safety benefits from a JAK1-selective strategy or alternative biologic therapies [24]. Current clinical protocols advocate for comprehensive pretreatment screening and continuous monitoring to ensure safe use [25].

JAK inhibitors versus adalimumab

The use of JAK inhibitors provides effective options for patients with moderate to severe PsA disease who may not respond adequately to standard DMARDs. Using JAK inhibitors in PsA has shown a valuable clinical outcome. JAK inhibitors and TNF inhibitors, like adalimumab, have demonstrated comparable efficacy. A head-to-head randomized controlled trial (RCT) revealed that upadacitinib (15 mg daily) was neither superior nor inferior to adalimumab (40 mg biweekly). Although upadacitinib at a higher dose (30 mg daily) yielded superior clinical responses, it was associated with more side effects, such as elevated liver enzymes and infection rates [17]. These findings highlight the importance of dose adjustment in balancing efficacy and safety.

Post-hoc analyses further support the role of JAK inhibitors in treatment sequencing. Patients transitioning directly from adalimumab to tofacitinib, as well as those maintained on tofacitinib, demonstrated comparable clinical efficacy and safety profiles [2]. While these findings suggest that tofacitinib may be a suitable alternative for patients who do not tolerate adalimumab, this conclusion is based on indirect evidence and should be interpreted with caution.

Some studies discussed the safety profile and adverse event rates of JAK inhibitors versus TNF inhibitors. A comparative study was conducted among many PsA patients and found no significant increase in cardiovascular (CVS) events or cancer among JAK inhibitor users compared to those on TNF inhibitors [26]. However, a post-hoc analysis of ORAL surveillance among RA patients revealed elevated cardiac risks among patients on tofacitinib (hazard ratio (HR) 1.98; 95% confidence interval (CI) 0.95 to 4.14) [27]. Therefore, regular follow-up and continuous monitoring are essential for the safety assessment of JAK inhibitors.

Serious infections are a common hazard with both JAK inhibitors and adalimumab. According to a cohort study, tofacitinib versus selective JAK inhibitors and TNF inhibitors are both linked to viral reactivation, specifically herpes zoster. However, TNF-α inhibitors are associated with aggressive infections, such as candidiasis and tuberculosis (TB) [17,28]. Moreover, a national real-world cohort study assessing the nonmelanoma skin cancer (NMSC) risk found comparable HRs with no significant difference in NMSC risk between the two medications. These results indicate that JAK inhibitors increase NMSC risk compared to the general population; however, JAK inhibitors are not associated with a higher risk of NMSC than TNF inhibitors [29].

TNF inhibitors have a greater drug persistence rate in PsA patients, although JAK inhibitors have a lower discontinuation rate. Disease activity and gender, more so in women, are additional factors that raise the likelihood of discontinuation [30]. Unlike TNF inhibitors, JAK inhibitors are small synthetic non-protein molecules; therefore, anti-drug antibodies and efficacy loss will not happen [31]. A comparative summary of JAK inhibitors versus TNF inhibitors is presented in Table 4, highlighting key differences in efficacy, safety, and clinical application.

**Table 4:** Comparison of JAK Inhibitors and TNF Inhibitors in Psoriatic Arthritis Management JAK: Janus kinase; TNF inhibitors: tumor necrosis factor inhibitors; PsA: psoriatic arthritis; HR: hazard ratio; CVS: cardiovascular system; NMSC: nonmelanoma skin cancer; TB: tuberculosis; STAT: single transducer and activator of transcription Credit: This table was created by the authors using data from multiple published sources to compare JAK inhibitors and TNF inhibitors in psoriatic arthritis. References show which study supports each data point. Citations are placed in each cell when different studies support each treatment arm. Risk estimates are reported carefully; if the confidence interval includes 1, we note there's no statistically significant increase in risk​​​​​​.

Factor	JAK inhibitors (e.g., tofacitinib, upadacitinib)	TNF inhibitors (e.g., adalimumab)
Mechanism of action	Targets intracellular JAK-STAT pathways	Block TNF-α, reducing inflammation
Efficacy	Comparable to TNF inhibitors at standard doses, superior at higher doses (but with more side effects) [2,17]	Comparable to JAK inhibitors [2,17]
Drug persistence	Lower discontinuation rate overall [30]	Higher drug persistence rate in PsA [30]
Immunogenicity	No risk of antidrug antibodies [31]	Can develop anti-drug antibodies, leading to secondary efficacy loss [31]
Cardiovascular risk	No statistically significant increase in CVS adverse events risk (HR 0.977; 95% 0.632, 1.510) [26]	No statistically significant increase in CVS adverse events risk [26]
Infections	Higher risk of viral reactivation (e.g., herpes zoster) (HR 2.37; 95% CI 2.00-2.80) [17,28]	Higher risk of aggressive infections (e.g., TB, candidiasis) (HR 0.25; 95% CI 0.09-0.73) [17,28]
NMSC risk	No statistically significant increase in NMSC risk HR 1.9 (95% CI 0.7 to 5.2) [29]	Comparable NMSC HR 2.1 (95% CI 0.8 to 5.3) [29]

JAK inhibitors versus IL-17/IL-23 inhibitors

In addition to TNF inhibitors, IL-17 and IL-23 inhibitors represent key biologic classes in PsA management. While much of the research has focused on comparing JAK inhibitors with TNF inhibitors like adalimumab, it’s also important to understand how JAK inhibitors compare with other biologic classes, especially IL-17 and IL-23 inhibitors, which are widely used in PsA.

When comparing JAK inhibitors to biologic DMARDs, several findings emerge. Overall, JAK inhibitors demonstrate comparable efficacy to biologic disease-modifying antirheumatic drugs (bDMARDs) regarding musculoskeletal outcomes, including joint tenderness, swelling, and composite disease activity scores [32]. However, IL-17 inhibitors and IL-12/23 inhibitors are preferred over TNF inhibitors in PsA patients with severe skin disease, according to the EULAR recommendations [32]. In contrast, JAK inhibitors, specifically tofacitinib at a dose of 5 mg twice daily, appear less effective than bDMARDs in achieving optimal skin responses [32].

Data from the PRO-SPIRIT study provides additional insight into early treatment responses. At the three-month mark, JAK inhibitors demonstrated rapid improvements in joint activity, including tender joint count (TJC), swollen joint count (SJC), and clinical Disease Activity Index for Psoriatic Arthritis (cDAPSA), compared with IL-23 inhibitors, which showed a slower onset of action [33]. However, when considering minimal disease activity (MDA) in patients with baseline body surface area (BSA) ≥3%, IL-17 inhibitors, particularly ixekizumab (IXE), achieved numerically higher MDA rates than JAK inhibitors [33]. Notably, secukinumab 300 mg (SEC300) demonstrated lower response rates compared with IXE and SEC150, likely due to more severe baseline disease in the SEC300-treated population [33]. These findings suggest that, while JAK inhibitors provide a faster onset of efficacy in joint disease, IL-17 inhibitors may offer superior skin responses in patients with more extensive psoriatic involvement.

Regarding safety profiles, IL-17 and IL-23 targeted agents generally exhibit a favorable risk profile. Data from clinical trials and post-marketing surveillance indicate a low risk of TB reactivation with IL-17 inhibitors, ustekinumab, and selective anti-IL-23 monoclonal antibodies [32]. Despite encouraging data, direct head-to-head comparisons across broader patient populations and longer follow-up periods are needed to fully clarify the safety and efficacy profiles of JAK inhibitors relative to other biologic DMARDs.

Clinical guidelines for PsA management

Major PsA guidelines position JAK inhibitors as second-line targeted synthetic DMARDs after conventional synthetic DMARD (csDMARD) or TNF inhibitor failure, with agent choice determined based on individual risk. EULAR (2023) and the ACR support JAK inhibitors for persistent high disease activity, recommending pretreatment cardiovascular, thrombotic, and malignancy screening and favoring JAK1-selective agents in high-risk patients [34]. GRAPPA highlights JAK inhibitor efficacy across all PsA domains but cautions those with thrombotic or serious infection histories and calls for ongoing monitoring of herpes zoster, lipids, and liver function [35].

Dosing and monitoring

To strike a balance between efficacy and safety, JAK inhibitor prescriptions should be patient-centered and closely monitored. Efficacy data from clinical trials show that filgotinib 200 mg, upadacitinib 15 and 30 mg, and tofacitinib 5 and 10 mg have improved ACR response rates, enthesitis resolution, and patient-reported outcomes in PsA [36,37]. For example, a comparison between tofacitinib 5 mg and 10 mg twice daily has shown slightly higher ACR 20 response rates with the 5 mg dose (50%) versus the 10 mg dose (47%), compared to 24% with placebo (P<0.001) [2,36].

The safety profiles vary across JAK inhibitor subtypes and doses. Tofacitinib 5 mg and 10 mg may have been associated with a modest decrease in hemoglobin (Hb) baseline during the third month of the trial (-0.2 and -0.3 g/dL, respectively) [13]. According to more recent data from RA populations, upadacitinib 30 mg may also cause a transient decrease in Hb and neutrophil counts, though these findings have not yet been confirmed in PsA [38].

Since the JAK inhibitors carry a potential risk of serious adverse events, including cytopenia, infections, and hepatic effects, pretreatment screening is essential. This includes a review of medical history and routine blood tests such as complete blood count, renal and liver function tests, hepatitis B virus status, and TB screening. Treatment should be individualized, considering each patient's preferences, medication cost, the presence of additional comorbidities, and disease severity. These recommendations are based on clinical trial protocols and expert consensus and aim to mitigate risks while managing disease severity [31].

Limitations

This narrative review has several limitations. First, while multiple JAK inhibitors were identified, the representation across JAK subclasses was unequal. Most available data focused on pan-JAK inhibitors (e.g., tofacitinib) and JAK1-selective agents (e.g., upadacitinib), while TYK2 and JAK3-selective inhibitors were underrepresented. This limits the generalizability of findings to newer or more selective agents.

Second, although some high-quality RCTs were included, many studies had small sample sizes, short follow-up durations, or relied on post-hoc analyses rather than pre-specified objectives. These factors introduce potential bias and reduce the strength of conclusions.

Third, heterogeneity in efficacy outcomes across trials, such as the use of ACR20/50 versus PASI75/90, reflects differences in study design, outcome measures, and patient populations. This variability made direct comparisons challenging and may affect interpretability.

Since JAK inhibitors are a relatively new class of medication for PsA, they have limited long-term safety, particularly regarding cardiovascular risk, malignancy, and venous thromboembolism. Finally, it is unlikely to completely rule out publication bias because studies with positive results can have a higher chance of being published, which could influence the perception of safety and efficacy in general.

## Conclusions

In the short term, JAK inhibitors appear to be effective in treating PsA's articular and dermatological symptoms, especially in patients unresponsive to conventional therapies. Trials involving upadacitinib, tofacitinib, and filgotinib have demonstrated improvements in ACR response rates, enthesitis resolution, and quality-of-life scores, with dose-dependent variations in benefit. While selective JAK1 inhibitors may offer a more favorable safety profile compared to non-selective agents, this observation is based on limited data and should be interpreted cautiously.

Safety concerns, such as infections, thrombotic events, dyslipidemia, and malignancy, vary by agent, dose, and patient profile. Most available data is short-term, and long-term safety outcomes remain uncertain. These risks should be evaluated in context, considering both the strength of evidence and clinical relevance. To optimize patient outcomes, individualized treatment plans and regular monitoring are essential. Future large-scale, head-to-head trials comparing JAK inhibitors with TNF and IL-17 inhibitors are needed to evaluate long-term safety, radiographic progression, and patient-centered outcomes such as quality of life.
